# Joint Energy Supply and Routing Path Selection for Rechargeable Wireless Sensor Networks

**DOI:** 10.3390/s18061962

**Published:** 2018-06-17

**Authors:** Liangrui Tang, Jinqi Cai, Jiangyu Yan, Zhenyu Zhou

**Affiliations:** State Key Laboratory of Alternate Electrical Power System with Renewable Energy Sources, North China Electric Power University, Beijing 102206, China; tlr@ncepu.edu.cn (L.T.); yabjy@ncepu.edu.cn (J.Y.); zhenyu_zhou@ncepu.edu.cn (Z.Z.)

**Keywords:** rechargeable wireless sensor network, network lifetime, energy supply, routing path selection

## Abstract

The topic of network lifetime has been attracting much research attention because of its importance in prolonging the standing operation of battery-restricted wireless sensor networks, and the rechargeable wireless sensor network has emerged as a promising solution. In this paper, we propose a joint energy supply and routing path selection algorithm to extend the network lifetime based on an initiative power supply. We develop a two-stage energy replenishment strategy to supplement the energy consumption of nodes as much as possible. Furthermore, the influence of charging factors on the selection of next-hop nodes in data routing is considered. The simulation results show that our algorithm effectively prolong the network lifetime, and different demands of network delay and energy consumption can be obtained by dynamically adjusting parameters.

## 1. Introduction

### 1.1. Background and Motivation

With the rapid development of information and communication technology (ICT), device-to-device enabled cellular networks [1,2] and machine-to-machine communication [3], wireless sensor networks (WSNs) have been widely deployed in various application scenarios such as volcanic eruption monitoring [4,5,6], power grid operation monitoring [7,8,9,10] and bridge health monitoring [11,12,13], etc. These applications have a common requirement for the lifetime of the sensor network, that is, the network can only work continuously and effectively for a certain time. The reason behind this fact is that it is extremely inefficient or even infeasible to perform frequent maintenance or change batteries in the abovementioned scenarios. However, the total amount of energy that can be carried by a sensor node is generally constrained by a variety of practical factors such as the limited physical space and battery capacity. Therefore, how to increase the network lifetime of WSNs is crucially important to guarantee reliable service delivery, and a subject which requires further investigation.

Currently, this field is receiving intensive attention from both academia and industry. The research results can be divided into three categories: energy-saving methods, energy-harvesting methods and initiative power supply method. Among the energy-saving method, the authors in [14,15,16,17,18,19,20,21] refer to reducing the energy consumption or workload per unit time of the sensor nodes by means of routing algorithm design, topological control, energy management, etc. These approaches generally maximize the network lifetime as the only goal. Besides, there also exists a research field where lifetime maximization is achieved by dividing redundant sensors into individually capable sets, and appropriately scheduling these sets [22,23,24,25]. However, as we have seen in [26], only optimising the lifetime through reducing energy consumption may be detrimental to the robustness of the network, and it just can prolong the network lifetime to a certain extent at the expense of certain network performance features, such as increasing the network delay or reducing the data reliability. The energy-harvesting methods [27,28,29,30,31] mainly convert some ambient energy such as solar, vibration, or wind in the surroundings into sensor node energy through an energy conversion module to extend its life. These methods are inexpensive and environmentally friendly, however, the conversion efficiency is not very high because of the low density of these energy sources, so the need to equip the sensor node with a large converter to obtain sufficient energy collection efficiency is unavoidable, which limits practical applications. The initiative power supply method is a kind of technology for wirelessly charging any sensor node by providing an active charging power supply in the network, such as laser active power technology [32], inductive coupling technology [33], electromagnetic radiation technology [34], and magnetic resonance coupling technology, etc. Among various potential technologies, the magnetic resonance coupling technology has received more extensive attention because of its good performance features, such as the high transmission efficiency [35,36]. The initiative power supply method has good controllability since specific sensor nodes can be selectively charged and power charged for these sensor nodes can be dynamically controlled. 

As mentioned above, each of these three methods has been object of many research efforts, but few studies have been done on the joint issues between them, and compared with renewable energy harvesting technology, the wireless energy transfer, which is based on magnetic resonant coupling, is able to provide more stable and reliable energy supply for the WSNs, so in order to improve the survival time of sensor network as much as possible and not limit real applications, a promising solution which combines the energy-saving routing method with initiative power supply method is proposed in this paper. Of course, the main problems and challenges include the following two aspects:

(1) Energy-saving routing algorithm design. Firstly, we need to design a reasonable forwarding node selection mechanism, and control routing cost as much as possible within a reasonable range. Secondly, how to predict the charging behavior cooperates with the energy supply strategy to maximize network utility and balance energy supplementation with energy consumption at full steam.
(2) Energy replenishment strategy design. Unlike the wired networks with approximately unlimited energy, the rechargeable WSNs require an optimized charging schedule to achieve permanent survival. If the initiative power supply method is adopted, the first question to be answered is how to allocate a limited amount of energy when the actual energy supply capacity is not sufficient.

### 1.2. Related Work

The topic of the energy shortage of wireless sensor networks has been already studied recently. Among these studies, in [37,38,39,40] the authors designed their respective routing algorithms and used energy-harvesting methods to replenish the energy for sensor nodes. In [37], the DEHAR algorithm finds all shortest path from each node to the base station using a directional diffusion routing protocol, and then updates the shortest path according to the energy state and distance penalty function of each node. The DEHAR algorithm is able to avoid low-energy nodes as much as possible, and allow them to gradually recover their power by capturing external energy. An energy-aware hierarchical topology control with energy capture technology for WSNs is presented in [38] to solve the uneven energy distribution issue. In [39], the authors propose a routing algorithm named energy harvesting aware ad hoc on-demand distance vector routing protocol (AODV-EHA). The AODV-EHA not only inherits the advantages of existing AODV, but also utilizes the energy harvesting capability of the sensor nodes to reduce the cost of transmitting data to the next hop. An adaptive energy harvesting aware clustering (AEHAC) routing protocol for WSNs is proposed in [40], which takes the energy state of nodes into cluster head election algorithm. All of these researches extend the network lifetime through the energy-saving routing algorithm and the energy-harvesting method, however, they have not taken into account the energy management of nodes brought by the randomness and uncontrollability of the environmental energy. Meanwhile, the routing only considers the remaining energy or the minimum transmission cost of the node, which is too one-sided.

The initiative power supply method has been studied to recharge sensor nodes in [41,42,43,44,45]. In [41], a periodical charging strategy has been designed to construct a shortest Hamilton Loop that takes the service station as the start and end points to traverse all sensor nodes. In [42], its strategy allows the wireless charging device (WCD) to move periodically along the traveling salesman circuit that contains all sensor nodes, and each sensor node will be fully charged before leaving. All of these studies assume that the energy supply capacity of a WCD is large enough to charge all nodes. However, restricted by the actual capability of WCD, charging all nodes may result in an overlong charging period, or some pivotal nodes cannot obtain relatively enough energy, which generates a low efficiency of charging energy utilization and inconspicuous impact on network lifetime extension.

In addition, there are some contributions in the literature that solve the issue of choosing nodes to be charged by setting a “threshold”. Among them, the authors in [43] sort the nodes from low to high according to their remaining lifetime, and set a target value of network lifetime. Under the condition of not exceeding the battery energy of the node and charging device, if a charging sequence that extends the network lifetime to the target can be found from the sorted list that contains the k shortest-lifetime nodes, then this algorithm updates the target network lifetime, and continues to the next loop until the maximum. In [44], multiple WCDs are employed to charge the sensor network, and the charging request will be automatically sent to the central dispatch node when the residual energy of the sensor node is below a certain threshold. A new MTER algorithm is presented in [45]. Firstly, they select those nodes below the warning energy line as the initial charging targets to prevent nodes from dying, then, each unselected node will be assigned a weight, and the higher the node weight value is, the more energy the network can acquire from the charging vehicle by means of selecting this node. These researchers’ works already can get extra network lifetime, however, when determining the charging nodes, they need to set an energy threshold directly or indirectly, which means that sensor nodes only will be charged when energy consumption reaches a certain level, and it is possible to shorten the actual network lifetime due to belated energy replenishment.

It is noticed that all abovementioned works simultaneously consider the routing and energy replenishment. However, most of these studies mainly focus on one aspect of the routing or energy replenishment, and some problems still exist in the energy supply process. Therefore, in this paper, we propose a new algorithm based on a joint consideration of routing and initiative energy replenishment, and considering practical problems, we study the limited energy supply capacity of WCD.

### 1.3. Contributions

Based on the above analysis, the major contributions of this paper are summarized as follows:

- We propose a two-stage energy replenishment strategy with limited energy supply capacity. The strategy uses an initiative power supply method with obvious advantages to charge the sensor nodes. Based on several important parameters such as the residual energy of nodes, the future energy consumption rate of nodes, the charging duration and charging speed of WCD, etc., each node of the charging time can be determined in two stages. Thereinto, we set a charging time update strategy to improve the energy utilization efficiency, which can optimize the energy distribution according to the WCD energy replenishment ability and the nodal energy consumption intensity.
- We propose an algorithm by considering the joint optimization of energy supply and routing. Jointly considering the energy status of nodes, energy consumption and energy supply, we propose a routing selection algorithm. In this algorithm, we consider not only the transmission energy from the current node to the next-hop node, but also the energy consumption of the next-hop node to the sink node, which impacts the delay and cost of data transmission. In addition, in order to avoid nodal premature death, the estimated residual energy of nodes has been taken into consideration, and the influence of energy supply model on the node transmission capacity has also been joined.
- We analyze the proposed algorithm from the perspectives of parameters and computational complexity. Besides, the algorithm is evaluated from the perspectives of the fusion index, harmonic coefficient, network size, charging duration based on our simulation. We compare the proposed algorithm with other two strategies—the proportional distribution strategy and greedy strategy. The simulation results show that our algorithm can effectively prolong the network lifetime, and it is able to obtain different demands of network delay and energy consumption by dynamically adjusting the relevant parameters.

The remaining parts of this paper are arranged as follows: Section 2 illustrates the rechargeable wireless sensor network of this article, at the same time, some formal definitions and concepts are presented. In Section 3, there is a detailed description of the proposed algorithm, and we provide the relevant algorithm performance analysis and implementation guidelines. The simulation results and relative discussions are presented in Section 4. Section 5 concludes the paper and briefly suggests some future research directions.

## 2. Guideline Supplements

**Figure 1:**
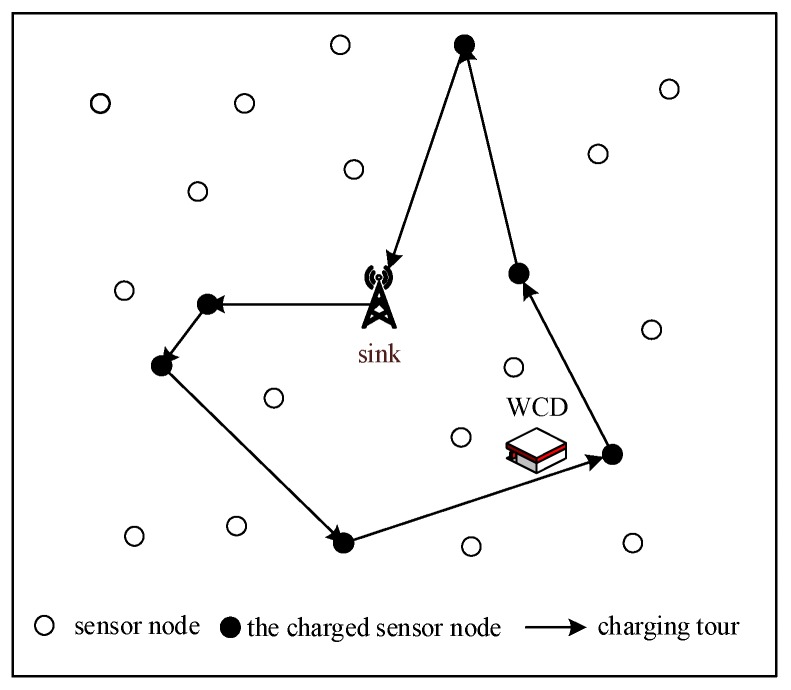
The rechargeable wireless sensor network.

Figure 1 shows the rechargeable wireless sensor network, which comprises a central base station (the sink node) that supervises the energy status of the whole network and formulates the charging schedule, a mobile charger carrying the WCD that is a programmable automatic aircraft with travel speed $v_{m}$, and n sensor nodes equipped with a wireless power receiver. The entire sensor network forms an undirected graph G(P,E), where *P* denotes the set of nodes and *E* denotes the set of edges.

We consider a sensor network with *n* nodes randomly deployed within a square area of area *S*, where the maximum communication radius of each node is configured to be *R*, and two nodes cannot establish communication links beyond this distance. Meanwhile, each sensor is equipped with a rechargeable battery with the same capacity $E_{0}$, and the node is considered as dead if it has a battery energy that decays to 0. Furthermore, the central location of the square area is selected as the sink node, which is responsible for summarizing and processing data.

The WSNs studied in this paper mainly focus on the typical data acquisition applications, in which sensor nodes periodically sense the relevant information in the monitoring area and transmit the monitoring data to sink node in a multi-hop manner. For purpose of facilitating further study, we need to simplify the sensor network model and give some reasonable assumptions as follows:

(1) The location of sensor nodes will not be changed after being deployed, and the distance from one sensor node to another can be estimated based on the received signal strength.
(2) All sensor nodes are isomorphic with the same function of routing, issuing and collecting, and they also have the same initial state. Besides, the residual energy of each node at any time is accessible to the base station, so that it can make decision. Moreover, the energy of sink is not limited.
(3) Each node collects data at an equal and constant rate r, and the sensed data is not aggregated with the received data. Besides, they adopt the first-in-first-out approach to packet processing, and the initial load is 0, the length of the cache queue BF is also limited.

Simultaneously, in order to describe the algorithm more clearly, our paper also makes the following relevant definitions.

**Definition** **1.** *The neighbor node set of node* i *is given by:*

(1) $$N(i)=\{j|d_{ij}\leq R\}$$

*where* $d_{ij}$ *is the Euclidean distance between node* i *and node* j.

**Definition** **2.** *The forward neighbor node set of sensor node* i *is* NF(i), *which can be expressed as:*

(2) $$NF(i)=\{j|j\in N(i),d_{j,sink}\leq d_{i,sink}\}$$

**Definition** **3.** The network lifetime refers to the persistent period from the deployment of the network nodes to the appearance of the first dead node due to energy exhaustion.

**Definition** **4.** *The forward distance* $d_{f}(i)$ *of node* i *is equal to the distance of node* i to the sink node, namely:

(3) $$d_{f}(i)=d_{i,sink}$$

**Definition** **5.** Charging capability is a measure of the amount of energy the sensor node receives from the charging device WCD per unit time.

**Definition** **6.** *The energy supply capacity is the measure of the amount of energy that the WCD can replenish the network during a charging duration* $T_{c}$. *Here, the charging duration represents the sum of the charging time of all charged nodes in a charging schedule.*

## 3. A Joint Algorithm of Energy Supply and Routing Selection

In this section, we introduce the joint algorithm of energy supply and routing selection for rechargeable wireless sensor networks. Firstly, according to the energy supply capacity of the WCD, the residual energy of the nodes and the intensity of energy consumption, how to allocate energy to the sensor nodes is described. Then, we will explain how to perform routing under the condition of charging. Finally, we analyze the theoretical properties and discuss the relevant implementation guidelines.

### 3.1. Two-Stage Energy Replenishment Strategy

In rechargeable WSNs, for the sake of achieving satisfactory performances and sufficient network lifetime, it is necessary for the researchers to consider the related problems and design a reasonable energy replenishment strategy which cooperates with the routing.

Firstly, the existing approaches mostly adopt mobile devices that can be applied to charge wirelessly all sensor nodes by moving along a certain path. However, the energy that the mobile device can carry at a single time has a certain ceiling in practical applications, that is, the energy supply capacity is limited, and so it is usually impossible and inappropriate to charge the entire network. When forcing the energy of all nodes to be replenished, it is likely that only a small amount of energy is obtained by each node, but it is hard to effectively delay the rapid energy decay of those dying nodes. Here, we consider nodes with low battery energy and/or high energy consumption as those dying nodes, or more precisely, these nodes are more likely to die from exhaustion. Secondly, after a period of operation of the network, the residual energy of each node can reflect the energy consumption rate of the node to a large extent, that is, generally speaking, the lower the residual energy is, the greater the energy consumption of the node, and vice versa. And when a node has many descendants, the remaining energy is usually lower due to the large amount of data forwarded, which means that there is a higher probability of death at the node during subsequent operations. Therefore, this subset of nodes should be the primary targets when considering the network energy supplement, and the remaining energy of sensor nodes will also affect how much energy each node obtains.

Therefore, an energy replenishment strategy based on limited energy supply capacity of the WCD is proposed in this section. The strategy uses a single WCD to recharge the nodes from the start to the end of the sensor network operations, so that the network can get more energy. What’s more, for the sake of replenishing the network as much as possible, the WCD should transfer all the limited energy it carries to the network, namely, there is a constant charging duration $T_{c}$ under a fixed charging speed in this work. Firstly, taking into account the remaining energy of each node and the capacity of WCD, the charging time is allocated according to the proportion of the maximum chargeable time of each node (i.e., the time it takes to fully charge the battery). Secondly, according to a defined time update strategy, the charging time of each node is readjusted so that more energy is concentrated on the nodes with the most serious energy consumption, and the effectiveness of energy replenishment and energy utilization are improved. It should be noted that we do not consider the one-to-many charging scenario, and the wireless charging device can only charge one node at the same time. Specifically, our charging scheme is as follows.

The initial energy of node i is $E_{0}$, the current remaining energy is $e_{i,t}$, and the charging speed of WCD is $\Lambda_{c}\eta$, then the theoretically maximum charging time can be denoted as:

(4) $$t_{i}=\left(E_{0}-e_{i,t}\right)/\Lambda_{c}\eta$$

where $\Lambda_{c}$ is the energy consumption of WCD’s charging operation, η represents the efficiency of converting and storing wireless energy, so $\Lambda_{c}\eta$ denotes WCD’s charging rate.

Next, as mentioned above, the current energy level of node i, which is equivalent to the maximum chargeable time, should play an important role in allocating the charging time. Therefore, the charging time in the first stage is preliminarily computed as:

(5) $$t_{i}^{\prime }=\frac{t_{i}}{\sum_{i=1}^{N}t_{i}}\cdot T_{c}\;$$

where $T_{c}<\sum_{i=1}^{N}t_{i}$, this is because our paper focuses on the charging strategy with limited energy recharge capability, that is, we think the WCD cannot supplement the battery ceiling for all nodes, and it is also consistent with the actual situation. At the same time, we assume that WCD’s charging rate for each node is a constant, so in order to reflect the limited energy capability, the value of $T_{c}$ is given in our charging schedule.

With a certain total charging duration, the charging time of each node can be determined according to Equations (4) and (5), however, it is difficult to maximize the network lifetime just by this. According to the definition in Section 2, the network lifetime refers to the death time of the first node, and the node with lower energy level in each round will get closer to death, so the network lifetime finally will be most likely decided by these nodes. Besides, in the case of insufficient WCD energy supply capacity, if we ignore this fact that some nodes with sufficient remaining lifetime during a certain period of time actually do not need or only need a small amount of energy supplement, and allocate the energy in accordance with the ratio of the maximum chargeable time, then, eventually, nodes with insufficient remaining lifetime cannot improve their energy level as much as possible, and WCD’s limited energy also fails to achieve a better effect. Therefore, this article further proposes a novel time update strategy to optimize energy allocation.

After the first phase of dividing the charging time, each node corresponds to an element ($N_{i},t_{i}^{\prime }$) where *N_i_* denotes a node numbered i(1≤i≤n), and $t_{i}^{\prime }$ denotes the original charging time of node *N_i_*. In a round of charging duration, the energy that can be added to the network is also limited, so in order to prolong the network lifetime, it is necessary to supply as much energy as possible to the fastest node in energy consumption, that is, we need to adjust the original charging time. As shown in Algorithm 1, the final charging time can be obtained by the following time update strategy:

- The strategy takes the mean value of historical data of each node i for a period of time as the future energy consumption rate ${\hat{c}}_{i,t}$, then calculates the remaining lifetime $l_{i,t}$ for all nodes and sorts them from low to high to receive an element set that needs to be updated:
  (6) $$M={\left\{\left(N_{i},t_{i}^{\prime }\right)\right\}}_{i=1}^{n}$$
  where M is the set of elements with $l_{i,t}<l_{i+1,t}(1\leq i\leq n-1)$. Then, it defines two pointers, that is k and m, whose initial values are set to 1 and n, respectively, pointing to the first element and the tail element of the set M.
- Taking into account the WCD’s limited energy supply capacity and the efficient usage of charging energy, the algorithm needs to adjust the original charging time. Firstly, when the charging time of node $N_{k}$ is $t_{k}^{\prime }+t_{m}^{\prime }$, and if the battery capacity ceiling has not been reached, then all the charging time of node $N_{m}$ is cut to node $N_{k}$ while the pointer m forward displacement of one, namely this pointer plus 1; otherwise, we cut part of $t_{m}^{\prime }$ to node $N_{k}$ to make it full of energy, and the pointer k backward displacement of one (that is, the pointer k minus 1). The algorithm continuously performs this step until the values of pointers k and m are equal or the remaining lifetime of node $N_{m}$ is less than ∂∗T.

**Algorithm** **1:** The Charging Time Scheduling Strategy**Input:** the initial energy $E_{0}$, the residual energy $e_{i,t}$, the estimated energy consumption rate ${\hat{c}}_{i,t}$, the charging duration $T_{c}$ of WCD, the charging rate $\Lambda_{c}\eta$, the location of each node. **Output:** each node’s charging time $t_{i}^{\prime }$ and the travel path of mobile charger. $t_{i}\leftarrow (E_{0}-e_{i,t})/\Lambda_{c}\eta$     /*the maximum charging time for each node*/ $t_{i}^{\prime }\leftarrow (t_{i}/\sum_{i=1}^{N}t_{i})*T_{c}$    /*the original charging time of each node*/ $l_{i,t}\leftarrow e_{i,t}/{\hat{c}}_{i,t}$         /*the estimated remaining lifetime of each node*/ Calculate the charging schedule cycle T according to $T_{c}$ and the maximum travel time. Sort the remaining lifetime from small to large to obtain the element set $M={\left\{(N_{i},t_{i}^{\prime })\right\}}_{i=1}^{n}$. k←1,m←n 
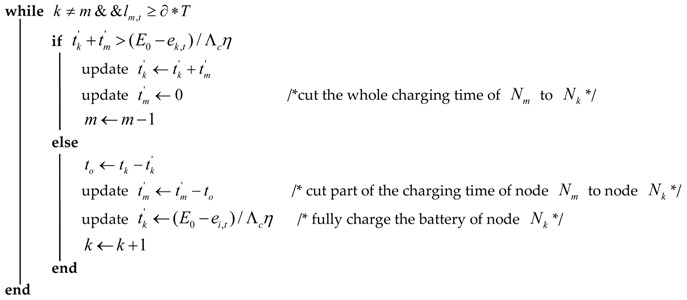
Calculate the shortest travel path for visiting nodes selected to be charged. **return**
$t_{i}^{\prime }$ and the shortest travel path.

Here, we must explain the above parameter ∂∗T. In our work, we provide the network with a periodic and continuous charging scheme to enhance the network lifetime. Based on this premise, it should be ensured that nodes which have not been charged in the current charging schedule have at least enough energy to spend in the subsequent rounds of scheduling time ∂∗T. In this work, we define the charging schedule cycle T as the sum of charging duration and the maximum travel time of the mobile charger, and the travel time of the mobile charger can be computed depending on the simulated annealing algorithm. The simulated annealing algorithm is a stochastic optimization algorithm based on the Monte-Carlo iterative solution strategy. Its starting point is the similarity between the annealing process of a solid material in physics and the general combinatorial optimization problem: starting from an initial temperature, with the constant decrease of temperature parameters, the global optimal solution of the objective function is randomly searched in the solution space by combining the probability jump characteristics, that is, it can jump out of the local optimal solution with probability and eventually tend to global optimum. Here, this algorithm is applied to finding a Hamilton circuit that contains all charged nodes, and the procedure is described in Algorithm 2. As for the maximum travel time, it should be reserved for the mobile charger to visit all the nodes selected to be charged in this round, so the maximum travel time is set up for the mobile charger according to a shortest Hamilton Loop that contains the whole network. 

**Algorithm** **2:** The Shortest Travel Path Based on the Simulated Annealing Algorithm**Input:** the flight velocity of mobile charger $v_{m}$, the coordinates C of all charged nodes in the current charging schedule. **Output:** the shortest travel path Ψ and the travel time of mobile charger $t_{m}$. Ψ←Ψ(i)     /*generate an initial path according to the coordinates C*/ L(Ψ(i))      /*the length of the current path*/ ℜ         /*used to control the cooling rate*/ Γ         /*the high initial temperature*/ Γ_min      /* the lowest temperature in the search process */ 
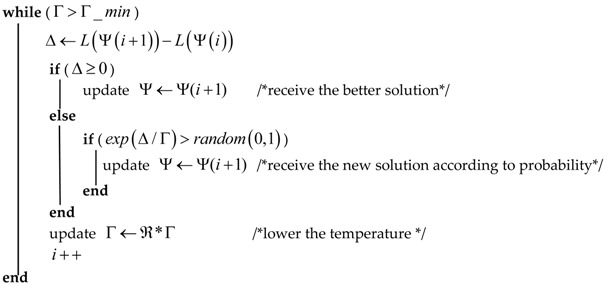
Calculate the travel time of mobile charger. **return**
Ψ and $t_{m}$.

Analysis of the charging algorithm shows that in the early stage of network operation, the total energy that the WCD can output during the $T_{c}$ time is generally more than the energy of the entire network deficiency, in this case, all nodes with relatively faster energy consumption can obtain sufficient energy, and the pointer *k* moves faster than the pointer m, and finally the update process will be halted when the two values are equal. However, as the network continues to run, nodes consume more and more energy, and the total energy that WCD can output during $T_{c}$ will not be enough to fill the entire network. In order to prolong the network lifetime, energy needs to be prioritized for nodes with more serious energy consumption. In our charging strategy, the fusion index ∂ determines the proportion of nodes that need to be prioritized when WCD’s energy is scarce, which affects the concentration of energy: the larger the ∂ is, the more energy the nodes will eventually be replenished, and the more decentralized the charging energy is; the smaller the ∂ is, the fewer energy supplements the nodes ultimately receive, that is, WCD replenishes other nodes after satisfying those nodes that have faster energy consumption and greater possibility of death. Thus, the charging duration and fusion index will have a significant impact on the performance of our algorithm.

### 3.2. Routing Selection

In WSNs, most sensor nodes cannot directly transfer data to the sink node by reason of the limited communication range, and the sensing data needs to be transmitted to the base station in a multi-hop manner through some relay nodes. Therefore, how to establish a data path from the source node to the sink node is the first consideration of any routing algorithm. In the traditional minimum energy routing strategy for saving energy and extend network lifetime, the transmission energy consumption to the next-hop node is minimized, so that the total transmission cost is the lowest. However, it is difficult to ensure that the energy consumption of subsequent transmission is minimized by only considering the minimum transmission energy consumption, and when sensor nodes are simultaneously on multiple energy minimum routes, their energy will be rapidly consumed, thereby accelerating the arrival of death. In addition, as we have described in Section 1.1, it is well-known that the minimum energy consumption which individually emphasizes the maximization of network lifetime is detrimental to network performances and a balance must be reached [26,46].

Therefore, for sake of a trade-off between the network lifetime and network performances, jointly considering the effect of the node’s state and our energy supply strategy, the routing weight of each forward neighbor node is defined in this paper. Meanwhile, we adopt the transmission energy consumption of current node i to the next-hop node j, the forward energy consumption of node j and the estimated energy of node i,j as the decision factors. After that, the candidate next-hop node with the smallest weight is selected as the actual next-hop node during the process of considering the route, and finally data packets are delivered.

Here, we define the weight of the connected edge E(i,j) from node i to its forward neighbor node j, that is, the routing cost of selecting node j as the forwarding node is:

(7) $$u_{i,j}=\alpha *\frac{C_{i,j}}{{\hat{e}}_{i,t}}+\beta *\frac{C_{j,s}}{{\hat{e}}_{j,t}},j\in NF(i)$$

where j belongs to the forward neighbor node set of current node i to avoid the return of data packets or forming a routing loop, $C_{i,j}$ represents the energy consumption of node i sending data to next-hop node j, and $C_{j,s}$ denotes the forward energy consumption of node j, both of which can be computed by the energy consumption model in [47]. ${\hat{e}}_{i,t},{\hat{e}}_{j,t}$ are the estimated remaining energy of nodes i and j (here, the node energy consumption during charging is ignored) after being charged, expressed as:

(8) $${\hat{e}}_{i,t}=e_{i,t}+\left(t_{i}^{\prime }-\gamma_{i,t}\right)*\Lambda_{c}\eta$$

In above equation, $e_{i,t}$ is the current remaining energy of node i, $t_{i}^{\prime }$ is the charging time allocated to node i, $\gamma_{i,t}$ denotes how long node i has been charged in the current $T_{c}$ interval. Here, considering the effect of energy supply on the energy level of nodes, and in order to balance the residual energy of each node at the end of each charging duration as much as possible to avoid the death of some nodes too early due to overuse, we adopt the nodal estimated residual energy instead of the current energy when defining the routing cost.

What’s more, the weight $u_{i,j}$ in Equation (7) expresses the common effect of the state of current node and the next hop node on the packet delivery, whose performances are affected by the relative sizes of the two. Note that, in this paper, we introduce harmonic coefficient α and β, which satisfy:

(9) α+β=1

From Equations (7) and (9), we can see that regulating the ratio of harmonic coefficient can achieve different network performances during routing selection. When α is larger, the state of current node i is mainly considered, whose transmission energy consumption and remaining energy after being charged will largely affect the choice of the next-hop node. At this point, the transmission energy consumption may decrease and the network hops increase. When β is larger, it strives to transmit data to the sink node within the allowable range in the fastest time, so that the number of hops is small but the energy consumption per hop may increase. Therefore, the dependency of data packets transmission on the two decision factors can be adjusted by changing α and β, which can achieve the expected network performances according to different requirements.

After obtaining the routing weight of each forward neighbor node, node i selects the node j with the smallest composite cost as the next-hop node, which is:

(10) $$j=\underset{k}{min}\left[u_{i,k}\right],\;k\in NF\left(i\right)$$

Finally, under the influence of transmission energy consumption, forward energy consumption of the next-hop node and estimated energy of sensor node, we can find all optimal routes to the sink for each node, and they are also the routes that have the potential ability of balancing the survival time and performances for the network.

### 3.3. Property Analysis and Implementation Guidlines

In this section, we will provide the relevant performance observations and analysis of our joint algorithm, and offer a simple implementation.

**Observation** **1.** *In the case of insufficient energy supply capacity of WCD, the fusion index* ∂ *can optimize the distribution of recharge energy and extend the network lifetime, and the smaller the value, the higher the energy utilization efficiency.*

According to Definition 6, the energy replenishment capability can be expressed as the product of the charging speed and the charging duration. In the case of one-to-one charging, this paper assumes that the charging speed remains unchanged, so the charging duration is equivalent to the size of the energy supply capacity. Thus, the charging duration is less than the sum of the maximum chargeable times of all nodes when the energy supply capacity is insufficient, so all nodes cannot be fully charged after the first phase of energy supply in Section 3.1, that is, there exists $\forall t_{i}^{\prime }<t_{i}$ for all i. It should be noted that the network lifetime is determined by the survival time of the first dead node, while the node that dies first usually belongs to those nodes with large energy consumption and large maximum charging time. Without the second phase of the energy supply strategy, the energy added to those nodes with low-load energy consumption is actually wasted after the death of the network, which seriously affects the utilization efficiency of limited energy. In the second phase, we define an update strategy which transfers the energy, which is originally distributed to those nodes with enough remaining energy, to others that needs energy urgently, so as to delay the death time of as much as possible, especially, when the fusion index is set to 1, that is, it must be ensured that the node can survive until the next round of charging schedule, the effect of prolonging the network lifetime should be the best.

**Observation** **2.** It is obvious that different harmonic coefficients can achieve different network performances.

The composite cost metric $u_{ij}$ for routing is defined in the proposed algorithm, and we also adopt the harmonic coefficients α to control the impact on the selection of next-hop node made by the transmission energy consumption $C_{i,j}$ and the forward distance energy consumption $C_{j,s}$. Among them, $C_{i,j}$ will certainly affect the final average energy consumption of network, and $C_{j,s}$ actually reflects the distance from the next hop node to the sink in some extent, which can affect the average number of hops. Thus, as it has been discussed in Section 3.1, we limit the sum of the harmonic coefficient α,β to 1, and the relative size of the harmonic coefficient reflects the bias towards energy consumption and hops during routing selection. Thereout, depending on different demands of the network hops and energy consumption in the real applications, the harmonic coefficient can be modestly changed. This is also confirmed and analyzed in the simulation part of Section 4.

**Observation** **3.** *In a worst case, the time complexity of our algorithm is* $O(n^{2})$.

The original charging time of each node is calculated in the first stage of the energy supply strategy, and the required time complexity is O(n). The first step in the second stage is to sort all the nodes by the merge sort according to their remaining lifetime, and the required time complexity is O(nlogn). The second step is to update the charging time of each node, and the worst case is that all nodes need to be updated, so the required time complexity is O(n). Therefore, the worst time complexity of the energy supply strategy is O(n).

The routing algorithm chooses one node from the forward neighbor nodes set as the next hop for each node. In the worst case, the number of forward neighbors of the node is n−1, so the worst time complexity is $O(n^{2})$.

In the process of solving the shortest travel path, The number of external loops under temperature control (i.e., the maximum number of iterations) is assumed to be a, and the number of internal loops at each specific temperature is b. Then, the time complexity of the entire algorithm is not O(a∗b∗n).

To sum up, the time complexity of our proposed algorithm in a worst-case scenario is $O(n^{2})$.

Next, we provide simple implementation guidelines of our algorithm. In order to prolong the network lifetime as much as possible, the algorithm burden borne by the sensor nodes should be minimized. Therefore, some tasks with high computing demands such as charging plan formulation are processed by the centralized computing infrastructure, while the routing selection can be executed locally. In addition, the load energy consumption of the nodes remains unchanged for a certain period of time. Therefore, it is possible to appropriately extend the communication interval that the ordinary nodes report their status to the sink node, so that reduce the communication energy consumption.

## 4. Simulation Results and Analysis

In this section, the proposed algorithm is verified by a large number of simulation experiments in Matlab, and we repeat each experiment many times to get an average. By analyzing the impacts of the fusion index ∂, the harmonic coefficient α, the network size *n* and the charging duration $T_{c}$ on the network lifetime, average hops and average energy consumption, it can be seen that our algorithm can commendably extend the network lifetime and improve network performances as we need. The average hops can signify the real-time nature of packets transmission to a certain extent, while the average energy consumption denotes the average total energy consumed in each data transmission cycle of all sensor nodes, can effectively evaluation the network energy efficiency. Relevant parameters adopted in our simulation are shown in Table 1. 

### 4.1. The Impact of Fusion Index on Network Performances

**Figure 2:**
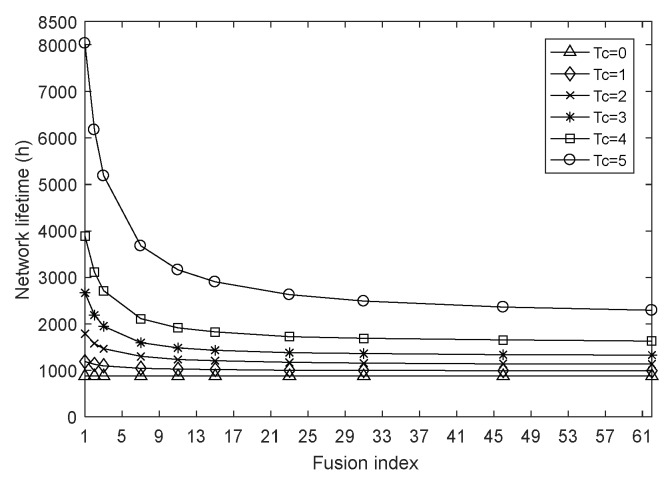
Network lifetime with varying fusion index.

**Figure 3:**
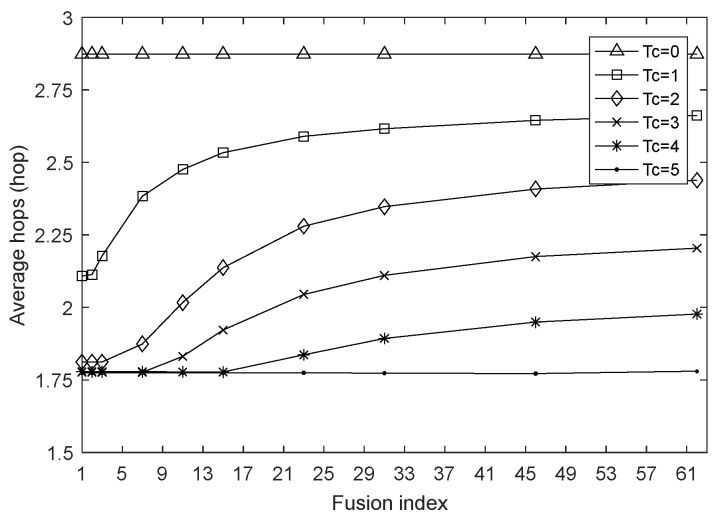
Average hops with varying fusion index.

**Figure 4:**
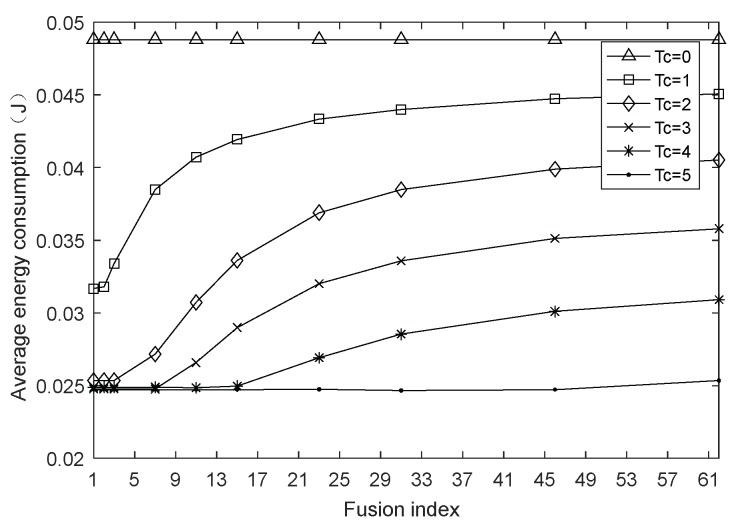
Average energy consumption with varying fusion index.

This section examines the impact of the fusion index on network performances by setting the network size to 100 and the harmonic coefficient to 0.4 and keeping them constant. Figure 2, Figure 3 and Figure 4 are the graphs of network lifetime, average hops and average energy consumption under varying fusion index and different charging durations $T_{c}$, respectively, where $T_{c}=0$ indicates that the network is not charged.

From the simulation results in Figure 2, the charging strategy presented in this paper significantly prolongs the network lifetime compared to the method of not using wireless charging for nodes, and in the late period of network, as the ∂ decreases, WCD charges nodes with low remaining energy more and more, that is, the closer the node to death, the more energy the node gets, so the effect of delaying node energy attenuation is more obvious, which further increases the network lifetime. For example, when the charging duration $T_{c}$ is 5, if the fusion index ∂ decreases from 15 to 1, the network lifetime increases by about 177%. In addition, it can be found that the impact of fusion index ∂ on the network lifetime is different. This is because the charge duration $T_{c}$ determines the total amount of energy that the network can obtain after one round of charging when the WCD’s charging rate has been determined, i.e., the size of the energy supply capacity, and the fusion index ∂ is able to concentrate energy and give priority to rapidly consuming nodes. However, when the charging duration is 1, the WCD’s supply capacity is so small that the energy gain of the node is very limited. Therefore, the decrease of the fusion index only makes the network lifetime slightly increase, which cannot achieve the fundamental change. But with the increase of $T_{c}$, the energy of WCD is relatively high, and the fusion index is more and more effective in optimizing the distribution of energy, accordingly, the network survival time is also longer.

Figure 3 shows the variation of average hops with the fusion index ∂. It can be seen that as the fusion index ∂ decreases, the limited energy can be more rationally distributed, and the node energy loss caused by data transmission can be replenished by WCD in time. Thus, in order to transmit the data packet to the sink node at a faster speed, the node will strive to maximize the single-hop forward distance so that the average hops of the network gradually decrease to ensure a tolerable network delay. Of course, we can see that with the increasing of fusion index, the growth rate of average hops is gradually decreasing and will eventually tend to be flat, because this paper not only considers the nodal energy, but also integrates the transmission energy consumption. Therefore, under the combined influence of these factors, the routing will be conducted in a most reasonable manner without unrestrainedly increasing the transmission distance, which demonstrates the good performance of our routing strategy.

From Figure 4, the charging obviously slows down the energy consumption rate of each node, moreover, it can be found that Figure 3 and Figure 4 have similar trends, because the average energy consumption refers to the sum of average energy consumption of all sensor nodes in each data transmission cycle over a period of time. As a major part of the nodal energy consumption, the transmission energy consumption in this paper is positively correlated with the square of the single-hop distance, and the average hops is inversely related to the single-hop distance in the case of the same network deployment. Therefore, when the number of nodes is constant, as the fusion index ∂ increases or decreases, the average hops presents a variation trend in Figure 3, at the same time, a similar trend in the average energy consumption with varying fusion index is found in Figure 4.

### 4.2. The Impact of Fusion Index on Network Performances

**Figure 5:**
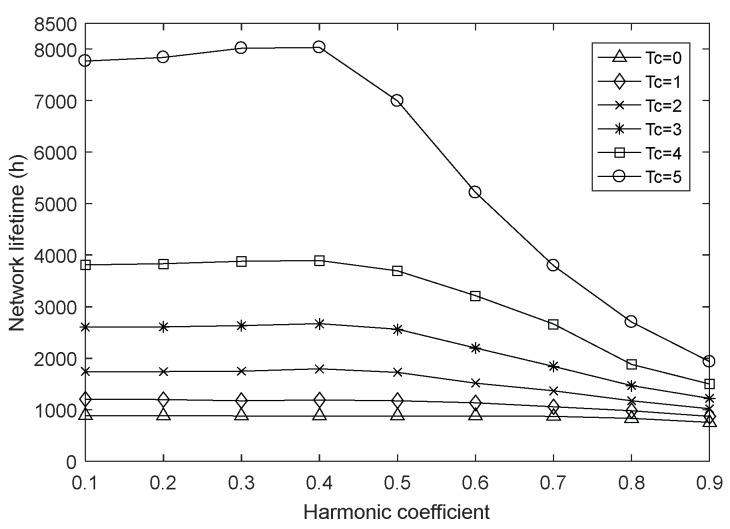
Network lifetime with varying harmonic coefficient.

**Figure 6:**
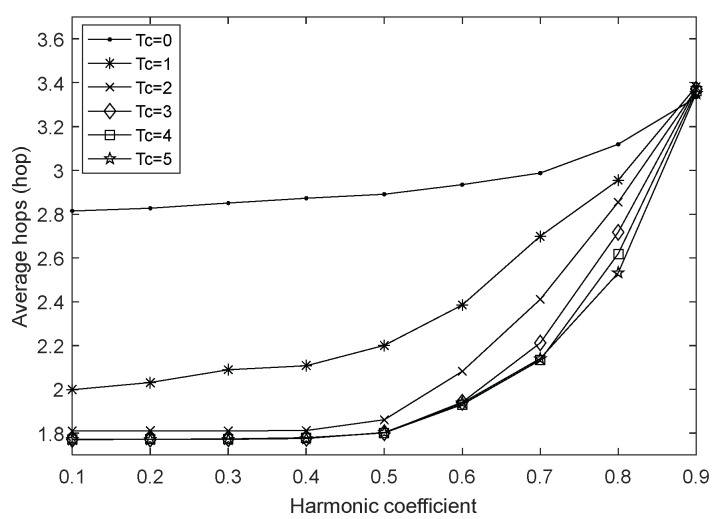
Average hops with varying harmonic coefficient.

**Figure 7:**
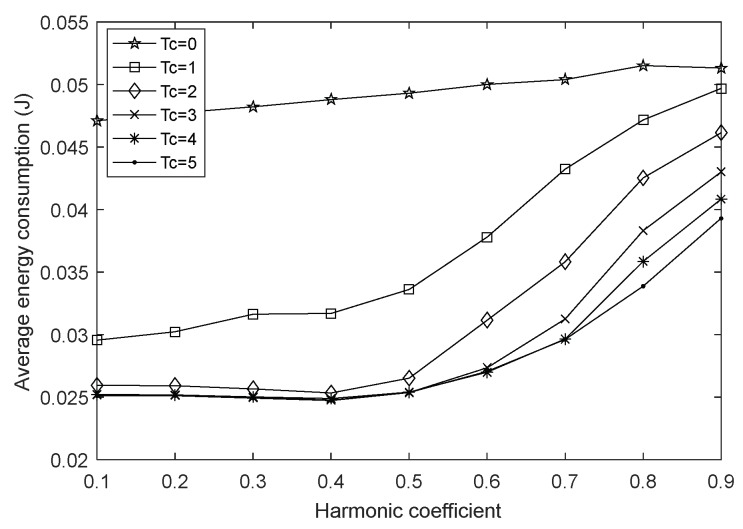
Average energy consumption with varying harmonic coefficient.

In order to explore the effect of harmonic coefficient *α* under different charging durations, we vary the value of α from 0.1 to 0.9 with a fixed step length, and set the fusion index to 1 and the network size to 100. The simulation results are shown in Figure 5, Figure 6 and Figure 7. 

As shown in Figure 5, the network lifetime increases slightly with the increase of α in [0.1, 0.4], but drops sharply with the increase of α in [0.4, 0.9]. This is because we take into account the transmission energy consumption from node i to node j, the forward energy consumption of node j and the estimated residual energy of both nodes, and the harmonic coefficient determines the degree of influence of the above factors on the next-hop node selection process. When the value of α is in [0.1, 0.4], with the increase of the harmonic coefficient, the selection of the next-hop node j is more and more focused on the state of node i itself, seeking the least transmission energy consumption and the largest estimated residual energy of node i, at this point, the forward distance of next-hop node may increases, but from Figure 7, it can be seen that the average energy consumption increases but the magnitude is not large when the charging duration is 1, basically unchanged or even reduced when the charge duration is 2–5, so the network lifetime can be extended. When the value of α is in [0.4, 0.9], as the harmonic coefficient continues to increase, the state of node j is excessively ignored when selecting the next-hop node j. As a result, the hop count of node i to sink increases greatly, resulting in greater energy consumption and making the network lifetime gradually reduced.

Figure 6 and Figure 7 show the variation of the average hops and the average energy consumption with different harmonic coefficients. Same as the Section 4.1, these two figures share a similar trend. Obviously, compared to the case of non-charging, charging the network allows the nodes to have enough energy and select the next-hop node with farther transmission distance, which can send data back to the base station as soon as possible, so there are less average hops after executing the charging strategy. Moreover, energy replenishment from the WCD results in less energy depletion per round, greatly extending the network lifetime.

In addition, it can be observed that the average hops and the average energy consumption increase with the increase of the harmonic coefficient when the charging duration is 0 (i.e., no charging occurs). This is reasonable as the fact that the value of the harmonic coefficient characterizes the importance of node i in routing selection at some extent. When α is increased, the forward distance of the next-hop node becomes larger and the average hops increases naturally. At the same time, nodes closer to the sink usually consume more energy, so the average energy consumption increases accordingly.

When the charging duration is 2–5 and the harmonic coefficient belongs to [0.1, 0.4], the average hops increases slightly with the increasing of harmonic coefficient. However, on the whole, due to the continual energy replenishment to the network, the average energy consumption has almost no change, but when α continues to increase within [0.4, 0.9], the influence of routing factors is unbalanced and unconscionable, so that the average energy consumption increases sharply while the average energy consumption also accelerates. Even when α is 0.9, the average hops exceed the value in an uncharged network, which is also evidence of the trend of the network lifetime in Figure 5. When the charging duration is 1, the WCD’s energy supply capacity is too small, and as α increases, in order to find the next-hop node with the lowest transmission energy consumption and the highest estimated residual energy, node i has to detour when transmitting data, therefore the average hops and average energy consumption have been increasing, and show a greater upward trend with the increase of α.

### 4.3. The Impact of Charging Duration on Network Performances

**Figure 8:**
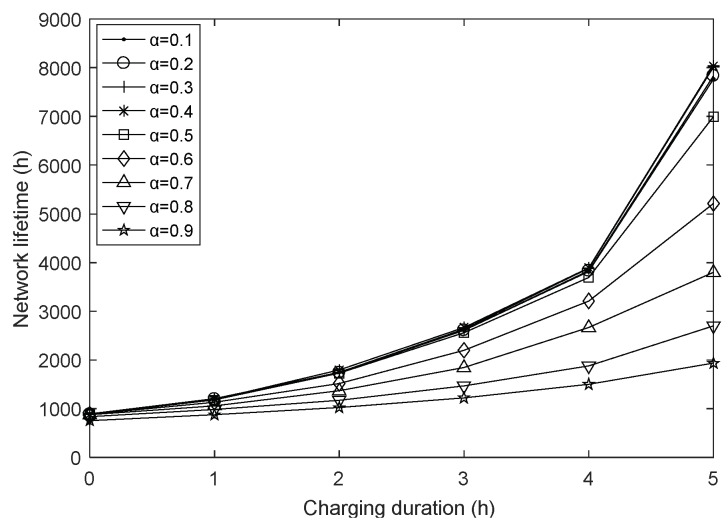
Average hops with varying charging duration.

**Figure 9:**
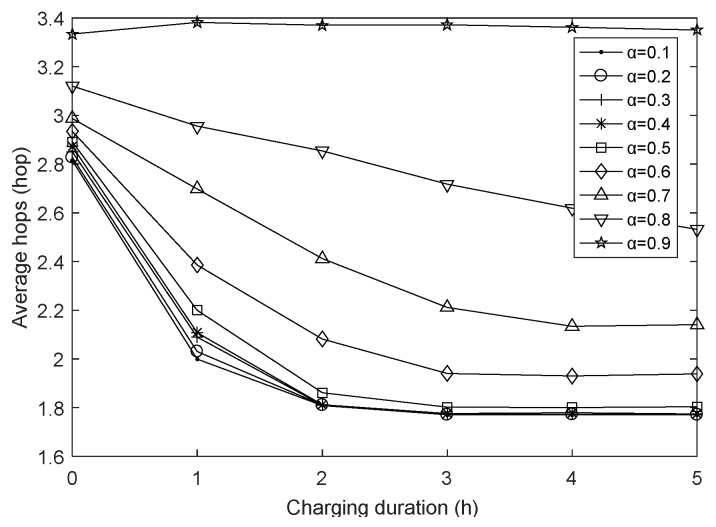
Average hops with varying charging duration.

**Figure 10:**
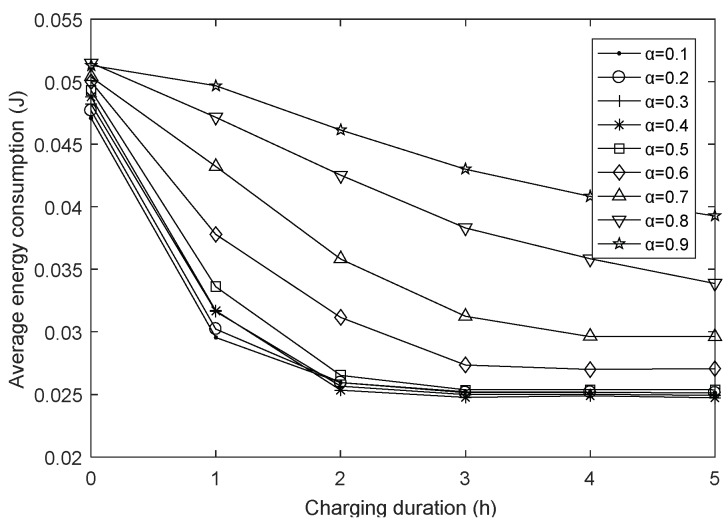
Average energy consumption with varying charging duration.

In this section, for purpose of studying the impact of charging duration $T_{c}$ more intuitively, we present Figure 8, Figure 9 and Figure 10 which are different visualisations of the data in Figure 5, Figure 6 and Figure 7. Figure 8 shows the network lifetime with varying charging duration under different harmonic coefficients. There is no doubt that the survival time of network will increase accordingly when we extend the total charging time. However, just like what has been presented in these three figures, the network lifetime with varying charging duration has different tendencies when the harmonic coefficient is in [0.1, 0.4] and [0.5, 0.9], and the best performance exists when it is 0.1. This is because, as it has been pointed out above, the minimum energy consumption routing is detrimental to the network performances. Besides, as it grows, metrics are increasingly biased towards energy consumption, so there is a trade-off between how we value the harmonic coefficient.

As it can be seen from Figure 9, the average hops decrease with the increase of the charging duration, thereinto, the average hops decrease significantly when the charging duration $T_{c}$ is in [0, 2], but the trend of decreasing is getting smaller and smaller when $T_{c}$ is in [2, 5]. This is because when the network is replenished with energy, the more the charging duration is, the more energy the sensor nodes can obtain. After getting enough energy replenishment, the routing can consider a forward distance of the next-hop node as small as possible, so the average hops decrease with the increase of the charging duration. However, since the maximum communication radius is $d_{0}$, the effect that the average hops decreases with the allowable communication range is obvious, but when $T_{c}$ continues to increase, constrained by the communication range and other routing decision factors, it is impossible for node i to pursue the single hop furthest indefinitely, and eventually the average hops change slowly. 

Figure 10 shows the effect of charging duration under different harmonic coefficients on the average energy consumption of the network, whose trend is very similar to Figure 9. Compared with the charge duration of 0, the decay rate of the network energy has been greatly improved due to the energy supplement of WCD. However, same as the Figure 9, the average energy consumption decreases with the increasing of the charging duration and the amplitude is not consistent. Specifically, on the one hand, it is related to the average hops, on the other hand, the limited energy supply capacity of WCD also plays an important role. In the early stage of network operation, the nodal energy loss is not serious, and the charging scheme can reduce the attenuation of the network energy well, but as the working time continues, more and more nodes lack energy, then the limited energy supply capacity can play a smaller role, and cannot significantly reduce the average energy consumption.

### 4.4. The Impact of Network Size on Network Performances

**Figure 11:**
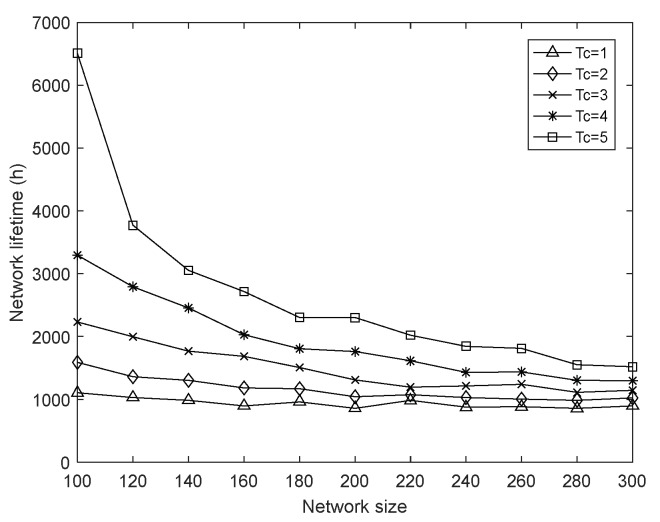
Network lifetime with varying network size.

In this section, we show the results of several simulations in the case of varying the network size n from 100 to 300, meanwhile we set the fusion index ∂ to 1 and the harmonic coefficient α to 0.4. Finally, the impact of node number on network lifetime is shown in Figure 11. It can be seen that under the same charging duration, the network lifetime gradually decreases under a decrescent slope when the network size increases. Meanwhile, when the charging durations are different, the impact of network size on the network lifetime is also discrepant.

In the WSNs, the “inner ring” nodes closer to the sink often consume energy faster than the peripheral nodes because of the more demand for forwarding data. When the network size gradually increases, more and more nodes need to send data to the sink node, which means that the heavier data forwarding task is undertaken by the “inner ring” nodes, and greater total transmission energy consumption exists in these nodes. Finally, it accelerates the emergence of the phenomenon of energy hole [48], that is, the “inner ring” node dies due to energy depletion, so the network lifetime becomes smaller and smaller as the network size expands. In addition, according to the charging algorithm in Section 3, the heavier the node’s task is, the more energy it can get during charging. Therefore, when the charging duration is continuously increased, the energy that the “inner ring” nodes can obtain gradually increases until they are fully charged, which can effectively prolong the network lifetime. Of course, as the network size continues to increase, the energy consumption rate of the “inner ring” nodes will eventually exceed the limited energy supply capacity of WCD, as a result, the network lifetime may increase with the charge duration, but the growth rate is getting smaller and smaller.

### 4.5. Comparision with Two Other Strategies

**Figure 12:**
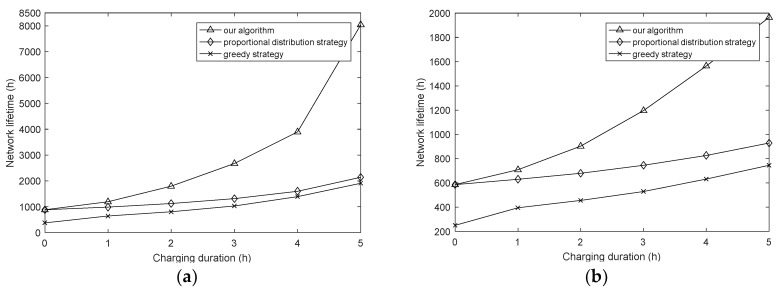
Network lifetime of different strategies with varying charging duration. (**a**) Packet size = 2000 bits; (**b**) packet size = 3000 bits.

**Figure 13:**
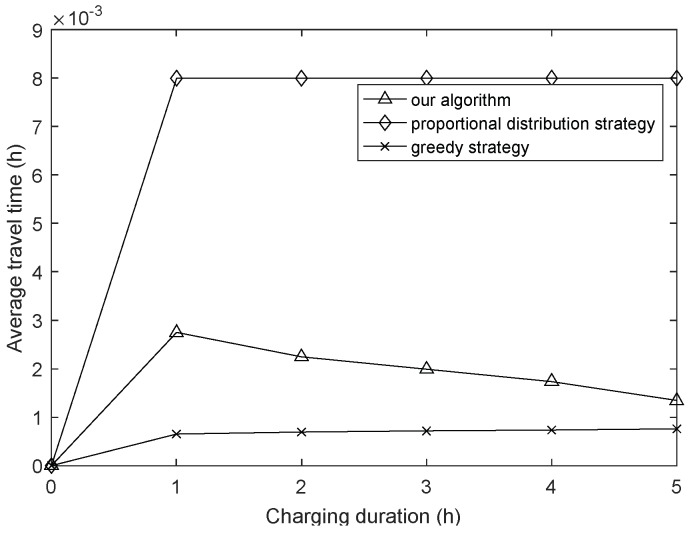
Average travel time of different strategies with varying charging duration.

In this section, we compare our proposed algorithm with two other strategies, that is, the proportional distribution strategy and greedy strategy. The results are show in Figure 12 and Figure 13, and the average travel time, which reflects the additional charging costs, represents the average time per charging scheme spent on traveling.

The proportional distribution strategy adopts the same routing algorithm as this article, while its charging strategy is only the first stage of our charging strategy, which means that this strategy needs to recharge the whole network. At the same time, the routing metric of the greedy strategy is the minimum transmission energy consumption, and its charging strategy is as follows: each sensor node sends a request message to the sink for charging, and then the WCD supplements the nodes with full energy from low to high according to the remaining lifetime until all nodes have been charged or the WCD’s energy has been exhausted. The greedy strategy method is adopted as the energy supply strategy in some references, such as [49,50,51]. By this way, we explore the contrastive result under different routing algorithms and energy supply strategies. It can be seen that although the average travel time of the algorithm in this paper is slightly higher than in the greedy strategy, it has the highest network lifetime, and the lifetime increases significantly with the increasing of charging duration, what’s more, the average travel time gradually decreased. Besides, in order to explore the influence of node task size, we test the network lifetime under two packet sizes, and the results are shown in Figure 12a,b. From the results, although the network lifetime is reduced due to the increased energy consumption, they have almost the same trend and we can conclude that the proposed algorithm is also suitable for different packet sizes under different task sizes.

## 5. Conclusions

To explore the routing and energy problems of rechargeable wireless sensor networks, a joint algorithm of the routing and energy replenishment based on a single wireless charging device with limited energy supply capacity is proposed in this paper. We not only establish a two-stage energy replenishment strategy which preferentially meets the energy demand of those nodes with faster energy consumption, but also consider a variety of factors, that include the charging, to define a routing selection metric of the next-hop node. The simulation results show that the algorithm can prolong the network lifetime effectively, and obtain different delay and energy consumption by adjusting parameters such as harmonic coefficient, fusion index and charging duration. In future work, we will focus on studying the cooperation of multiple wireless charging devices, in which the sensor nodes are mainly powered by an initiative power supply method and supplemented by an energy-harvesting method.

## Figures and Tables

**Table 1:** Simulation Parameters.

Definition	Notation	Value
Simulation area	S	$100\times 100\;m^{2}$
Network size Nodal original energy	n $E_{0}$	100~300 0.5 J
Maximum communication range Data rate	R r	30 m 8000 bit/h
Buffer size	BF	20 packets
Sink	(sink.x,sink.y)	(50 m,50 m)
Charging rate Charging duration Fusion index Harmonic coefficient Flight velocity of mobile charger	$\Lambda_{c}\eta$ $T_{c}$ ∂ α $v_{m}$	0.0045 J/h 0~5 h 1~62 0.1~0.9 30 m/s
